# Practical Review on Aetio-Pathogenesis and Symptoms in Pigs Affected by Clinical and Subclinical Oedema Disease and the Use of Commercial Vaccines Under Field Conditions

**DOI:** 10.3390/ani15152275

**Published:** 2025-08-04

**Authors:** Juan Hernandez-Garcia, Isaac Ballarà Rodriguez, Ramon Jordà Casadevall, Sergi Bruguera, David Llopart, Emili Barba-Vidal

**Affiliations:** 1 HIPRA UK and Ireland Ltd., Nottingham NG76LH, UK; 2 HIPRA, 17170 Amer, Spainsergi.bruguera@hipra.com (S.B.);

**Keywords:** Shiga toxigenic *Escherichia coli*, STEC, edema disease *Escherichia coli*, EDEC, Shiga toxin 2e, Stx2e, verotoxin, oedema disease, subclinical oedema disease, oedema disease vaccines, Shiga toxin vaccine, recombinant Shiga toxin

## Abstract

The impact of Oedema Disease produced by Shiga toxigenic *Escherichia coli* (STEC) in swine is increasing in some production countries due to increasing limitations on treatment with antimicrobials and zinc oxide, either because of the increased prevalence of multi-resistant strains or because of legal restrictions. The main pathological effect of Shiga toxin 2e is represented by damage to the endothelial cells of the blood vessel walls, leading to liquid extravasation and oedema formation in multiple tissues. These oedemas are generally easily identifiable in acute clinical cases. However, disease caused by Shiga toxin can occur without any externally visible oedema in the pigs, as observed in the subclinical presentation of Oedema Disease. It also causes productive losses, so it is important to identify and/or diagnose cases to set up control measures in order to optimize production and health. This article includes a comprehensive review of lesions and signs caused by Shiga toxin toxicosis in pigs, as well as other insights about the aetiology and epidemiology of STEC in pigs, and the effect of Shiga toxin recombinant toxoid vaccines in reducing these clinical and subclinical signs under field conditions.

## Simple Summary

Oedema Disease caused by Shiga toxigenic *Escherichia coli* (STEC) has a big impact on pig production. Shiga toxin 2e is the toxin that causes the pathological effect, which mostly causes damage to vascular endothelial cells and leads to oedema formation and productive losses that are easily detectable in its acute clinical presentation. The subclinical presentation of the disease does not present visible oedemas but there are lesions and productive losses. This article reviews different aetio-pathogenic aspects of clinical and subclinical disease produced by STEC with an emphasis on productive impact and the use of commercial oedema vaccines under field conditions.

## 1. Background

Oedema Disease causes significant economic losses for pig producers due to increased mortality, reduced weight gain and the additional cost of treatments [1]. It is produced by a group of *Escherichia coli* strains collectively known as “Shiga toxin-producing *E. coli*” or “Shigatoxigenic *E. coli*” (STEC), “Verotoxin-producing *E. coli*”, “Verotoxigenic *E. coli*” (VTEC) or “Edema Disease *E. coli*” (EDEC) with the alternative spelling for the word “edema”. All these terms—VTEC, STEC and EDEC—are used as synonyms in swine medicine for Stx2e-producing *E. coli* strains [1]. There are multiple types of Shiga toxins (produced by STEC strains) causing pathologies in humans, livestock and wildlife [2], but they have no known effect on pigs. Stx2e is the only one described to cause a pathology in swine [1].

Traditional approaches to the treatment of Oedema Disease have focused on management of the post-weaning digestive dysbiosis, trying to avoid the overgrowth of *E. coli* by implementing changes in nutrition management and formulation, and adding feed additives such as zinc oxide or using antimicrobials [3]. Other nonspecific measures focused on preventing and reducing the risk of colibacillosis include further nutritional measures, water quality control, feeding strategies, environment optimization, hygiene measures or control of concomitant diseases, among others; quality reviews and other literature provide further details of all these measures and their potential [4,5,6]. Unfortunately, these nonspecific measures only reduce risks of clinical disease and they cannot prevent colibacillosis or STEC overgrowth by themselves.

Farms with a history of Oedema Disease problems generally rely on prevention with antimicrobials and/or therapeutic zinc oxide (usually >2000 ppm zinc) in post-weaning diets. These measures have had a relatively high success rate in preventing clinical Oedema Disease outbreaks but a limited impact after the onset of clinical signs [1]. The risk represented by preventive treatments involving antimicrobials is of great concern, as there is evidence that the inclusion of antimicrobials in feed has contributed to the generation of multi-resistant *E. coli* strains worldwide [7]. Consequently, an increasing number of countries have modified their legal framework to reduce, limit or even ban these practices—i.e., in June 2022 the European Union banned the inclusion of zinc oxide in feed as a therapeutic product at levels over 150 ppm zinc. Colistin was an antimicrobial widely used to control post-weaning diarrhoea but its use was also restricted in Europe and other regions. These limitations, in combination with changes in the quality and source of feed ingredients—due to climate change and other geopolitical aspects affecting supply—have exerted a negative impact on intestinal health control of weaning pigs and increased the incidence of post-weaning diarrhoea and Oedema Disease.

The arrival of commercial vaccines with recombinant Shiga toxin subunits has created a new approach to prevention, which does not attempt to avoid the dysbiosis but instead focuses on neutralizing the Shiga toxin toxic effect, with a high rate of success in reducing the clinical impact of Oedema Disease and preventing mortality [8,9]. Vaccines have been reported to improve productive parameters in clinically and subclinically affected pigs by improving performance, reducing the prevalence of concomitant disease and decreasing antimicrobial use [10]. Vaccines have also contributed to making the subclinical impact of Oedema Disease more evident, as vaccination against Shiga toxin can exert significant productive improvement on STEC-positive farms without the presence of signs of clinical Oedema Disease [11].

This article is an up-to-date review of scientific publications and field data on STEC-caused disease pathogenesis, clinical signs and the use of Oedema Disease vaccines. This information on epidemiology, pathogenesis and treatment approaches is relevant for a better understanding of STEC; this has special relevance in regions where treatment options—antimicrobials and zinc oxide—are restricted, but it is also important at a global level in the context of rationalizing and efficiently using antibiotics.

## 2. Aetio-Pathogenesis of Shiga Toxin 2e and Lesions

Oedema Disease is produced by a group of *E. coli* strains initially characterized by its cytotoxic effects on Vero cells caused by the toxins they produce, hence the adoption of the name verotoxin for the toxin. Some researchers adopted the term Shiga-like toxins to describe the newly identified cytotoxins in *E. coli*, which exhibited similarities to *Shigella dysenteriae* toxins. Eventually, over a decade following the discovery of STEC, it became evident that Shiga toxins shared a common mechanism of action and similarities in genetic information. In 1996, an international panel of researchers agreed to designate this group of biologically similar toxins as Shiga toxins regardless of their bacterial origin, in recognition of the original discovery nearly a century earlier [12].

*E. coli* strains causing Oedema Disease have been traditionally characterized as alpha-haemolytic strains, containing F18 (F18ab) adhesin and Shiga toxin 2e (Stx2e), also known as verotoxin 2e (VT2e or Vtx2e) by other authors. Nowadays, the inclusion of an *E. coli* strain in the STEC group is not exclusive to the presence of these traits, so there are STEC strains that are non-haemolytic; F18 can be absent, and they can present other types of adhesins—i.e., F6, AIDA or eae intimins-, and some strains can produce additional toxins such as EAST1. In terms of O antigens—capsular lipopolysaccharides—O138, O139, O141, O147 and O157 have been reported [1,13,14,15].

STEC can colonize the swine intestine without causing pathology, but when the circumstances are favourable, it overgrows and produces Stx2e, which, after absorption, will produce its pathological effect. Pigs can carry STEC for life [16] or just for a period and even become non-detectable in clinically affected pigs by the time they die [17,18,19].

Stx2e is produced in the intestinal lumen or in STEC-invaded lymph nodes [1] and absorbed into the bloodstream, where it reaches its target cells in different locations of the body. When Stx2e is in contact with the target cells, it binds to specific surface receptors, preferably globotetraosylceramide (Gb4), but also globotriaosyleramide (Gb3) [19]. After binding, the toxin internalizes in the cytosol, producing a toxic enzymatic effect on ribosomes by inhibiting protein synthesis, which leads to cellular death by necrosis [18,20,21].

Endothelial cells in small blood vessels such as arterioles and capillaries are the main type of target cells for Stx2e, but other types of cells, such as myocytes, leucocytes and erythrocytes, can also be targeted [1,18]. Vessel tunica media myocytes that are affected by Stx2e undergo necrosis; this is a typical lesion that is observed altogether with accumulation of fibrinoid material on the vessel walls [21,22].

Some vessel segments can be more damaged than other contiguous segments, and large veins or arteries are not affected. Vessels draining tissues where the toxin is produced or absorbed—such as mesenteric venae and lymph nodes—are more likely to present the above-mentioned angiopathy. Stx2e’s predilection for causing more damage in some specific tissues has been reported in literature; some authors suggest it is due to the level of blood irrigation [21,23], while other studies have suggested it is linked to Gb4 receptors’ cell-surface expression levels [21], though no hypothesis has been proved.

Damage in endothelial cells produces a degenerative angiopathy leading to loss of functionality of the vessel lining; as permeability increases due to the insult, there is an extravasation—or leakage—of fluid from vessels, creating a non-inflammatory oedema in surrounding tissues. When the insult is large enough, cells—such as erythrocytes—escape the vessels, producing haemorrhages and microthrombi [20,23]. Further negative effects of Stx2e interfering with the immune system and immunomodulation have also been proposed [24,25,26].

**Figure 1:**
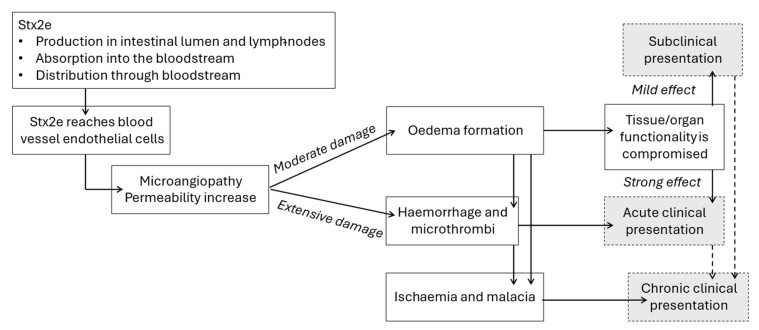
Toxin production and pathological effects producing different clinical presentations. Stx2e is produced by STEC in the intestinal lumen and/or lymph nodes, then absorbed into the bloodstream. Stx2e will produce its pathological effect on endothelial cells, causing increased microangiopathy and vessel permeability. Moderate pathological damage to vessels caused by Stx2e produces oedemas in surrounding tissues, which compromise tissue and organ function. Depending on the severity of this functionality loss, signs presented by affected animals are inapparent (or subclinical) when the effect is mild, or acute clinical when signs are evident. When Stx2e causes an extensive pathological damage, haemorrhages and microthrombi produce visible clinical signs (acute presentation). When lesions and poor tissue irrigation are maintained in time they can produce ischaemia and malacia. Oedemas, haemorrhages and microthrombi lesions can resolve and repair relatively fast and heal quickly, but once ischaemia and malacia lesions are produced, they will require more time if not chronic; a variable percentage of animals with clinical and sub-clinical signs do not successfully heal but develop chronic signs for a variable period of time.

Depending on which organs or tissues are affected and the severity of the lesions, a wide range of clinical signs are observed, from subclinical to severely impairing the animal’s health or even causing death in a very short period (Figure 1; Table 1). Only a few clinical signs and lesions associated with Oedema Disease or Stx2e toxicosis that are reported in the literature are easily detectable in field cases, while some others remain unnoticed. Mesocolon oedema is one of the hallmark findings at post-mortem, while the most relevant clinical findings are the occurrence of sudden death of the best performing pigs in the batch, eyelid and forehead oedemas, and neurological signs [1,21].

**Table 1:** Lesions described in the different tissues/organs and associated symptoms (continue on the next page).

Tissue/Organ	Specific Associated Lesions	Symptoms Associated with Loss of Functionality
Blood vessels, arterioles and capillaries. Large vessels are exempt.	Microangiopathy. Loss of continuity of lining. Necrosis of the tunica muscularis smooth muscle cells.	Oedema in surrounding tissues. Haemorrhages in surrounding tissues. Microthrombi. Ischaemia.
Central nervous system: Brain Cerebellum Brain stem Cervical spine Meninges	Angiopathy in vessels in meninges. Oedema in leptomeninges and perivascular space producing compression of the nearby structures, such as cerebrum and cerebellum, causing loss of functionality.	Seizures. Sudden death. Behavioural changes (Figure 2): Diminished consciousness Anorexia. Reduced water intake Low reactivity to stimuli and reduced activity General pain due to the oedema Pigs find relief in pressing foreheads against a wall Locomotor effect: Incoordination, dizziness Movement in circles Prostration
	Infarction of vessels producing ischaemia of tissues and malacia in chronic cases.	Postural changes, asymmetric stance leading to change in muscle conformation. Twisted head position.
Skin	Subcutaneous oedema.	Pruritus and swollen areas (oedema) that are visible in certain areas such as eyelids, forehead and chin (Figure 2 and Figure 3).
Ears	Microangiopathy. Reduced irrigation and necrosis.	Secondary bacterial infection. Large areas of necrosis on the auricular pinna (Figure 4). Loss of auricular tissue.
Lungs	Oedema. Patchy sub-lobular congestion.	Difficulty breathing due to lung oedema and reduced alveolar capacity.
Larynx	Oedema.	Altered phonation mechanism, changing pitch and vibration of normal squeak.
Stomach	Oedema in fundic and cardiac submucosa. Gastric haemorrhages. Ulceration.	Interrupted digestion: fresh dry feed is usually found at post-mortem. Presence of blood (from stomach) in intestinal contents.
Intestines and nearby tissues	Microangiopathy. Intestinal wall oedema Mesentery oedema; gelatinous mesocolon oedema, colonic submucosal oedema (Figure 5).	Altered functionality, intestinal permeability and nutrient absorption capacity. Small intestines are generally empty at post-mortem. Constipation is frequent, although diarrhoea can be observed during the clinical phase of the disease.
	Intestinal haemorrhages in most severe cases.	Blood contained in the faeces in cases where there are gastric or enteric haemorrhages. Pigs with these severe lesions die in most cases.
Pancreas	Oedema.	No specific signs reported.
Gallbladder	Oedema.	No specific signs reported.
Kidney	No damage reported.	No evidence of malfunction, contrary to other mammals.
Heart	Petechiae in epicardium and endocardium. Haemorrhages in myocardium. Liquid accumulation in pericardial sac with strands of fibre.	
Thoracic and abdominal cavities	Accumulation of liquids with strands of fibre.	Distended abdomen.
Eye (retina)	Oedema.	Impaired vision.

Adapted from [1,14,18,19,20,21,22,23,27,28,29,30].

**Figure 2:**
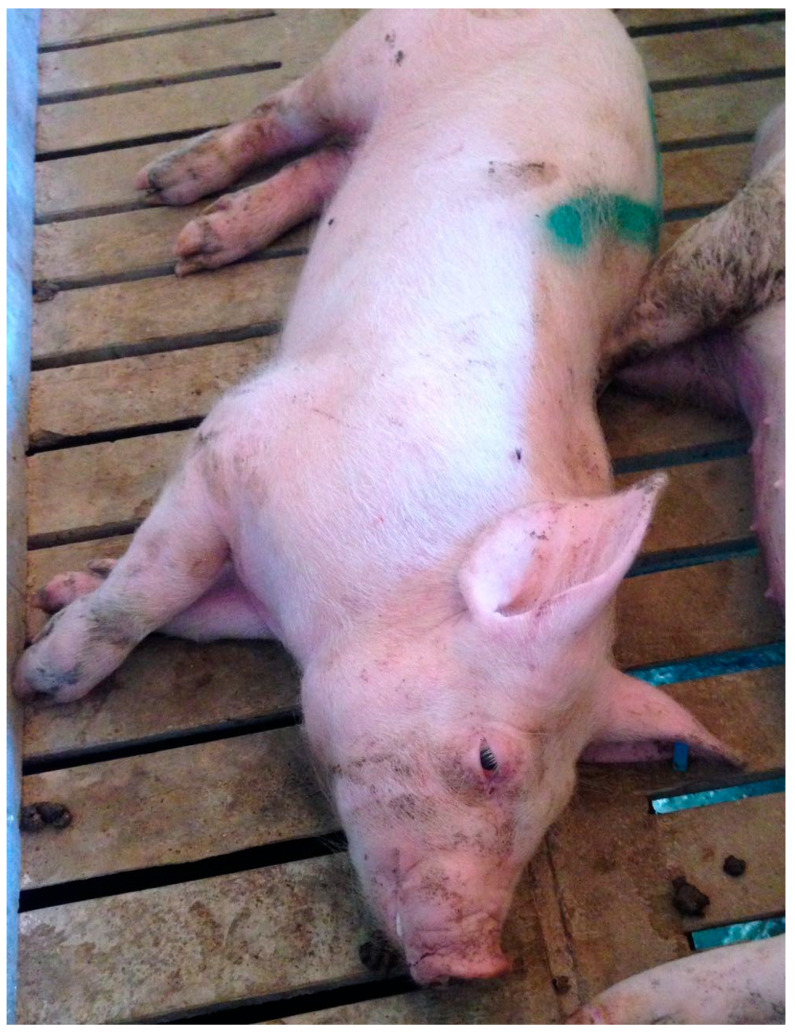
Good quality pig affected by acute Oedema Disease; subcutaneous oedemas were visible in eyelids. Behavioural changes were severe; consciousness and reactivity to stimuli were reduced. Credits: Hipra archive.

**Figure 3:**
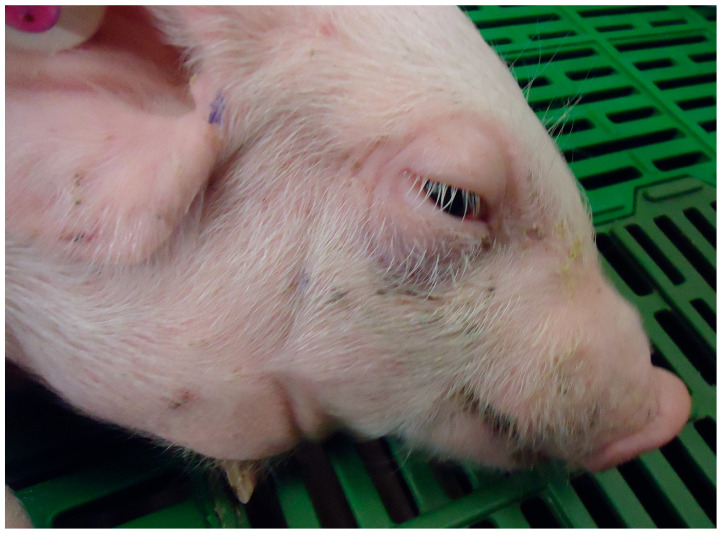
Pig with Oedema Disease. Subcutaneous oedema is noticeable in eyelids, snout, forehead and neck. Credits: Hipra archive.

**Figure 4:**
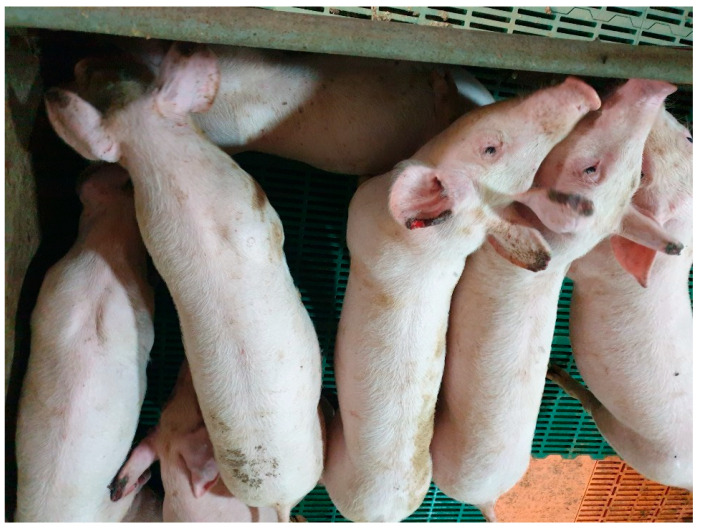
Ear necrosis cases observed in herd with an ongoing subclinical Oedema Disease problem. Credits: Hipra archive.

**Figure 5:**
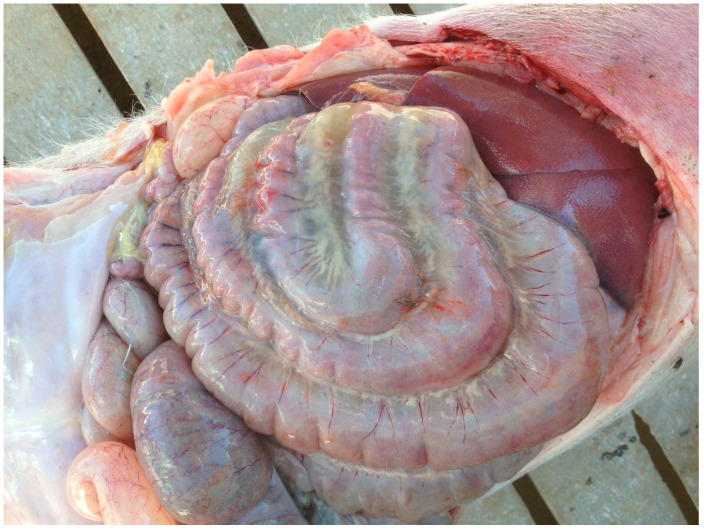
Gelatinous mesocolon oedema, which is considered a hallmark macroscopic lesion in fatalities caused by Oedema Disease. Credits: Hipra archive.

Neurological clinical signs caused by Stx2e toxicosis can be mistaken for other meningitis episodes, especially streptococcal meningitis [31], which is the most frequent cause of severe neurological episodes in pigs aged 4 to 16 weeks [32,33,34]. They show certain similarities and occur in pigs in the same age range, but there are some details concerning the clinical course and outcome that make it possible to differentiate between them. Severe cases of streptococcal meningitis can be accompanied by vestibular syndrome, neck rigidity and hyperextension of the head, paddling movements of the legs or lateral nystagmus [34], which are signs absent in cases of Oedema Disease or Stx2e toxicosis [1]. Also, Stx2e toxicosis does not cause fever [19] as a systemic bacterial infection such as *Streptococcus suis* would do [34]. Macroscopic and microscopic examinations of lesions in the brain leave no room for confusion due to the pyogenic nature of *Streptococcus* spp. producing purulent meningitis, which is easily detectable in the meninges, while Stx2e produces a much-reduced inflammatory response with angiopathy and oedema—sometimes also malacia—as the main lesions in brain and other tissues [20,21,34]. Further information regarding histopathological lesions and findings in field cases and typical lesions associated with the different presentations of Oedema Disease is available in Table 1 and Table 2.

## 3. Toxicodynamics, Toxicokinetics and Immune Response Against Stx2e

A dose-dependent pathological effect of Stx2e has been suggested based on in vitro experiences and other animal models [36,37]. Also, a time-dependent effect has been described in other mammals for several STEC Shiga toxin family toxins [38,39]. The effective time and dose of Stx2e producing a toxic effect in the target is modulated by additional factors such as the level of toxin production by a particular strain [40,41], the amount of toxin absorbed through the intestines or the possible presence of some active or passive immunity binding to the toxin and blocking its effect [18,20].

The relationship between dose and effect is not clear, as pigs inoculated with similar Stx2e doses showed different clinical presentations and degrees of lesions [20,21]. A study measuring Stx2e levels in STEC-infected pigs described how clinically affected animals had a higher surge of Stx2e on the 4–5 days post-STEC infection compared to the other animals without clinical signs. However, there was no difference in the levels of Stx2e in the blood of clinically affected pigs and pigs with no clinical signs on the following days; There is a clear lack of knowledge regarding dose, time and effect but the above-mentioned studies suggest that the outcome of an STEC infection also depends on some factors after Stx2e is absorbed into the bloodstream which vary at an individual level, making some pigs more susceptible to Stx2e than others [18].

These theories—of individual variation effect, dose-dependent effect and time-dependent effect—help to explain the variability in intensity and location of lesions and different clinical presentations [18,20,42] as detailed in this review (see Table 1). An additional factor modelling the effect of dose and time in Stx2e toxicosis is the presence of neutralizing antibodies. There are no very specific data available on the immune response against Stx2e, but naïve pigs can create neutralizing antibodies as soon as a week after exposure to Shiga toxin [43]; this contrast with recombinant Stx2e commercial vaccines datasheets define the period of onset of immunity as three weeks; though in field experiences with natural challenge some degree of protection is observed much shorter period, especially when animals were previously exposed to STEC by natural infection (from the authors’ personal field observations (unpublished)).

Anti-Stx2e neutralizing antibodies are very effective in preventing the Stx2e pathological effect and productive impact and they are the only commercially available effective strategy to block Stx2e damage once produced [1,44]. Recombinant Stx2e-based vaccines stimulate the production of anti-Stx2e neutralizing antibodies; they do not avoid STEC colonization or overgrowth, but the anti-Stx2e antibodies prevent mortality entirely and reduce clinical signs and the effect on productive performance to a minimum. These and other effects on concomitant diseases and reduction of antimicrobial use have been described in multiple trials comparing control groups and Stx2e-immunized pigs affected by clinical or subclinical problems (Table 3).

Maternally derived immunity can also play an important role, as sows in infected herds can present Stx2e-specific antibodies that are found in colostrum and the offspring, which decrease by the time of weaning [83] and increase after natural challenge [84]. Attempts to vaccinate sows with Stx2e recombinant vaccines to transfer immunity and protect pigs in the 2–3 weeks following weaning have been reported in the literature [20,85] but such protection is not observed or reported in field conditions. Maternally derived anti-Stx2e neutralizing antibodies are lost soon after weaning [86] and that is the period of higher risk to suffer Oedema Disease problems; however, it has the potential to cause problems in older stages as STEC is found present in the pig intestinal microbiota for several months after weaning [16].

## 4. STEC Infection Dynamics and Prevalence

There may be knowledge gaps about how STEC affects pigs at an individual level, but there are no fewer gaps concerning epidemiology at the population level. There are no studies detailing how STEC infection spreads between different pigs in a group, such as a pen or barn, or different age groups in the same farm. Weaners can become infected in the growing phase if not already positive when they were in the breeding unit. Outbreaks and clinical disease do not depend only on the infection status—and prevalence—at weaning but more on triggering factors. It is not uncommon to observe that different batches of weaners from the same breeding unit (site 1) that are sent to different nurseries/growing facilities (site 2) only suffer clinical Oedema Disease during the growing phase when going to a particular site 2, while they stay clinically “healthy” at the other site 2 farms (own unpublished data).

Although the causes that can trigger a clinical outbreak of Oedema Disease in a herd are not clearly defined, the necessary condition is an overgrowth of STEC, which can be triggered by—for example—the transition to solid feed at weaning, a change of diet in the growing period, a sudden increase in feed protein levels, resistance to antimicrobial treatments. Any factor leading to a change in the intestinal microbiota has the potential to trigger an outbreak in an STEC-positive herd [1,19,87].

The prevalence of STEC-infected herds in the different production regions is unknown. A recent study carried out on voluntary samples collected for screening or monitoring purposes from 3785 farms in 47 countries detected the presence of the STEC Stx2e gene in 62.75% of the herds [88]. Prevalence was high and constant in the different regions: 61% in Europe, 64% in America and 66% in Asia. These data agree with previous studies estimating prevalence ranges of between 30% and 60% of farms carrying STEC [89,90]. Expression of the F18 receptor on pig enterocytes is necessary for STEC colonization; F18 receptors are not expressed in newborn piglets’ enterocytes, but most pigs express them by the third week of age and it is still present at the age of slaughter [91]. There is a positive correlation between age and STEC detection [16]. STEC was shown to be able to persist in pigs for an extended period of time after initial infection [1,25].

## 5. Common Disease Presentations Associated with STEC

Clinical signs caused by STEC most commonly develop in pigs in the first 2–4 weeks following weaning, at approximately 4 to 12 weeks of age [1,19,92]. Digestive disorders can occur concurrently, as during this period the intestine undergoes serious modifications in structure and microbiota in order to adapt to the new post-weaning diet while maternally derived immunity wanes; this represents a major challenge for the digestive system and is when problems can occur [93]. These digestive disorders are not necessarily related to STEC but other *E. coli* (Enterotoxigenic *E. coli*, Enteropathogenic *E. coli*, Enteroaggregative *E. coli*) or other pathogens such as *Salmonella* spp., different types of *E. coli* or Rotavirus, among others [94,95]. Some STEC strains have the potential to cause diarrhoea but that is not a common feature. In non-acute cases in which pigs continue eating, some looseness and diarrhoea can be observed as a product of the disruption of the normal architecture of the small intestine, leading to poor absorption of nutrients and secretion of fluids to the gut lumen [21].

As a dose-dependent toxicosis, different types of clinical signs will be produced; peracute, acute, chronic and subclinical presentations have been described in the literature at the individual level [1,20,21,22,42]. Each clinical presentation has a series of lesions and symptoms that are more common for that presentation (Table 2). However, these clinical presentations only apply on an individual basis. A group of pigs represents a population, and not all of them are necessarily infected with STEC at the same time and the toxicosis can progress at a different pace, so it is common to find different clinical presentations during a Stx2e toxicosis outbreak, ranging from peracute disease to “apparently healthy” subclinical presentations and from acute cases with sudden deaths to chronic disease presentation.

The percentage of pigs displaying each type of clinical presentation can vary greatly and can quickly change over time, especially with the peracute and acute presentations.

### 5.1. Peracute Presentation

The peracute presentation occurs with sudden onset followed by death in 8 to 12 h [20]. Clinical signs can be so subtle that they can pass unnoticed; nervous signs characterized by prostration, locomotor impairment and breathing problems reflect the effect of the toxin on the cerebrum and cerebellum [1,20]. Prior to the disease onset, most of these pigs commonly present good performance and have a higher feed intake than the other pigs in their batch. Famers and stockpeople frequently fail to detect the signs, so they only report the number of suddenly dead pigs that can exceed 15% of the pigs in the pen in a day [1].

### 5.2. Acute Stx2e Presentation

The acute presentation is the most frequently described and is easier to detect as it includes the whole range of signs produced by the oedemas, such as laryngeal oedema, producing a squeaky grunt, externally visible oedemas mostly on the eyelids and forehead, difficulty in breathing and nervous signs [19,20]. Nervous signs include locomotor and behavioural changes such as impaired movement, tendency to low activity and prostration and anorexia, whilst some pigs find relief by pressing their foreheads on a wall. Pigs with a lower intensity of clinical signs can recover, but once the neurological signs develop, the most frequent outcome is an increase in severity followed by death in the next 24–48 h, despite veterinary treatment [1]. Recovery takes several days for the acute clinical signs to disappear at the individual level—oedemas disappear relatively quickly once the recovery has started-, and up to a week at herd level if properly treated [19]. However, a mid-to-severe loss of growth potential—measured as average daily weight gain—is reported in some of these animals, not only during the convalescent period but it has also been observed in pigs that have apparently recovered for several weeks after the event.

After an acute episode, lesions can decrease in intensity and become subclinical or heal, but some lesions can persist for longer, causing ischaemia and evolve into a chronic presentation (Figure 1).

### 5.3. Subclinical Presentation

The subclinical presentation has been described by different authors [1,18,20,21]. There are no criteria for individual in vivo diagnosis; some authors have reported a decrease in growth rate at the group level [30,35] while more individualized studies were also able to report lesions associated with subclinical disease [20,21,30].

In trials exposing pigs to Stx2e, animals both with and without clinical signs were susceptible to the development of lesions caused by Stx2e [20,23]. Immunization against Stx2e in subclinically affected pigs has a positive impact on improving performance indices and reducing antimicrobial use and concomitant diseases, such as problems related to *Streptococcus suis* or ear necrosis (Table 3)—though further evaluations are necessary to properly describe the mechanism leading to this effect. This emphasizes the relevance of the subclinical presentation and the benefits of mitigating its direct and indirect (through concomitant diseases) impact on herd health.

### 5.4. Chronic Stx2e Presentation

The chronic presentation is found in animals that have recovered from acute disease but have developed some ischaemic lesions in locations such as nervous tissue and muscles, causing some loss of function that takes weeks to recover. These animals can present impaired growth for weeks and some nervous problems affecting them unilaterally, such as movement in circles, asymmetric stance, with a twisted position of the head. After some time, they can also develop muscular atrophy and weakness in the limbs [1]. It is not clear if the subclinical presentation can progress to a chronic presentation but the lesions of microthrombi and haemorrhages are also reported in subclinical cases [20,21]. It is expected that the extent and location of the ischaemic area have an impact on the intensity of sequelae.

## 6. Concurrent Diseases and Misdiagnosis

Diarrhoea is sometimes found concurrently with clinical or subclinical Oedema Disease; there are descriptions of *E. coli* strains containing genes of other toxins in addition to Stx2e—such as heat stable and heat labile toxins—[1,96]. Actually, some of these strains are labelled as ETEC as they are more prone to produce an enterotoxic effect [1]. STEC proliferation in the intestine can be promoted by a dysbiosis but can also contribute to producing it [97]; it has been suggested that the effect of Shiga toxins on enterocytes enhances *E. coli* colonization [98].

In cases of Oedema Disease, there are detailed descriptions of different types of intestinal lesions affecting gut integrity, structure and functionality [20,27] that increase intestinal lining permeability and can produce a process known as Leaky Gut Syndrome (LGS) in which nutrient absorption is impaired and toxins and bacteria pass from the intestinal lumen to the bloodstream [99]. Further studies are needed to provide better prove and description of how this happens, but this process would be a rational explanation for the different studies in which they found certain types of connections between STEC, Oedema Disease and additional problems caused by *Streptococcus suis* in pigs during the weeks following weaning (see Table 3); as translocation has been described as a common method for bacteria such as *Streptococcus suis* to reach the bloodstream of swine and humans [100,101], a LGS can be a facilitator to enhance this sepsis.

**Figure 6:**
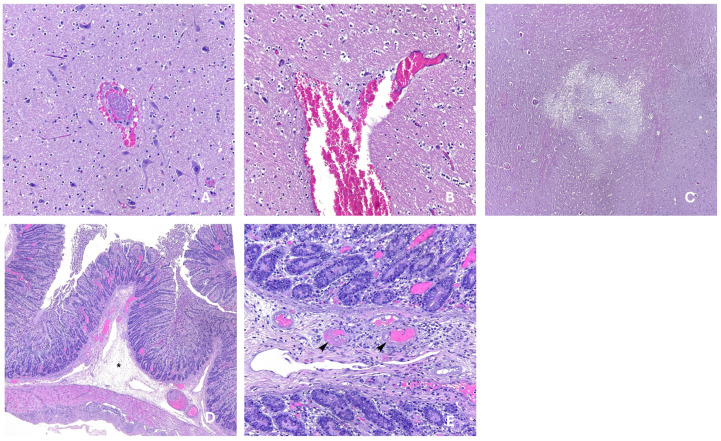
Histopathological images from a case of subclinical Oedema Disease [31]. (**A**) Thrombus and perivascular haemorrhage in the central nervous system (200× magnification); (**B**) Perivascular haemorrhage in the thalamus; (**C**) Focal spongiosis in the central nervous system (100× magnification). (**D**) Colonic submucosal oedema—lesion marked with asterisk—(100× magnification); (**E**) Fibrinoid degeneration, necrotizing vasculitis and thrombosis—lesion marked with arrow heads—in venules of colonic mucosa (200× magnification). Credits: UAB (Autonomous University of Barcelona) Veterinary Pathology Diagnostic Service.

Certain similarities in the neurological clinical signs between Oedema Disease and *S. suis* are a reason for common diagnostic failures, but they can act concurrently as described. An example of a case involving both was finally diagnosed as subclinical Oedema Disease; implementing vaccination with Stx2e reduced the average mortality from 7.4% to 4.3% [31]. Stx2e vaccination in piglets also ended the neurological signs that this farm was observing in the nursery phase, which were mostly related to vestibular syndrome, which started to be seen at the same time as mortality increased. This was a known STEC-positive farm without Oedema Disease-like signs and the initial tentative diagnosis was *S. suis* infection as the causative agent. Macroscopic observations at post-mortem did not offer any other insights and *S. suis* was isolated from samples. The emergency antimicrobial treatment—amoxicillin, chosen based on antimicrobial susceptibility test results—had no effect in reducing clinical signs. Results from histopathology (Figure 6) revealed thrombi and perivascular haemorrhages together with spongiosis in central nervous system tissue. The colonic submucosa showed oedema and necrotizing vasculitis. These lesions were compatible with the pathology produced by Stx2e and vaccination proved to be effective in reducing the problems.

Future controlled challenge studies are required to better understand the type of interactions caused by Stx2e and *S. suis*; whether it is an immunomodulation effect, increased translocation or some other mechanism, there are cases in which Oedema Disease prevention helps to reduce the impact of other concomitant diseases.

Another pathology that has been suggested to be possibly associated with Oedema Disease is ear necrosis. To our knowledge, there are no peer-reviewed articles associating ear necrosis as a direct result of the pathological effect of Stx2e; however, a reduction in the incidence of ear necrosis was reported in trials applying vaccines that generated opsonizing antibodies against Stx2e [11]. Ear necrosis is known to be a multifactorial problem with other pathogens such as *Treponema pedis* or *Staphylococcus hyicus* being frequently recovered on those lesions [102]. The role of these bacteria is subject to discussion and in some cases it can be defined as opportunistic; in the case of STEC, the hypothesis is that the vascular injury produced by Stx2e—characterized by segmental changes within the tunica media and presence of karyorrhectic nuclear debris—could lead to poor regional irrigation, tissue death, extended necrosis and opportunistic infections in the poorly irrigated or dead ear pinnae tissue. Other hypotheses link ear necrosis with the LGS and intestinal dysbiosis as a product of a “Swine Inflammation and Necrosis Syndrome” (SINS). SINS is a newly identified distinctive syndrome consisting of different microbe-associated molecular patterns (MAMPs) generated in one part of the body but that can influence other areas due to the activation of the immune system at a general level rather than locally [103,104] The intestine is an organ with particularly intense contact with bacteria among other pathogens and molecules with toxic effects which represent a source of stimuli to the development of SINS; and SINS has been described as another factor contributing to ear necrosis [105] among others previously described such as genetics, diet, management and environment [105,106].

## 7. Discussion and Conclusions

Oedema Disease in swine presents a wide variety of signs and lesions with clinical, subclinical and chronic presentations in which visible lesions and oedema are not necessarily present. The terms “Shiga toxin Associated Syndrome” and/or “Shigatoxicosis” (Stx2e toxicosis) would better define and represent the disease produced by STEC.

As production losses occur in the absence of clinical oedema signs, it is important to detect Stx2e toxicosis or identify those signs and/or lesions associated with it. This would be the basis for a diagnosis in an ongoing case. Detection of STEC would be enough to classify the farm as infected and implement preventive measures to avoid problems. Risk factors leading to dysbiosis that can trigger an outbreak—i.e., feed modifications, protein level increase, ingredient change—should be taken into consideration and properly monitored. Also, STEC-positive farms should be periodically monitored for the presence of subclinical disease; however, there are no diagnostic criteria by which to investigate the presence of subclinical disease. Due to difficulties in establishing a proper diagnosis of subclinical disease and the even more difficult task of evaluating its incidence and productive loss, vaccination trials including control groups and measuring basic key performance indicators—such as mortality rate or weight gain—are a practical, cost-effective way to evaluate the impact of subclinical disease.

STEC-positive farms should consider adopting a conservative approach when introducing changes in nutrition, management or other factors with the potential to cause dysbiosis, as this can trigger an Oedema Disease outbreak. Training farm staff and pig stockpeople to rapidly detect and report the early signs of disease is important to mitigate the impact in the case of an outbreak.

There are still substantial gaps in our knowledge regarding this disease. Some of these areas are the molecular and physiological mechanisms of Stx2e, or a more detailed understanding of the subclinical presentations. From a practical perspective, a more accurate economic evaluation of management strategies, risk factors and preventive measures—such as the use of vaccines—would help in the prevention of Oedema Disease. Control strategies involving dysbiosis risk reduction, such as limiting or reducing protein contained in feed, limiting the feed offer or replacing local protein sources with higher quality protein and/or amino acids have the potential to produce a negative impact on growth performance and/or feeding costs [107,108,109]; it is important to explore the different options for maintaining health at the highest possible level with no detriment in terms of productive and economic results.

In terms of disease prevention, antimicrobial treatments may provide short-term solutions but should only be used during new outbreaks in emergency situations. These treatments should be replaced by preventive measures as soon as possible. Commercially available vaccines are highly effective in controlling the challenge of Oedema Disease; the main limitation of these vaccination protocols is that maternally derived antibodies do not protect pigs after weaning—and Oedema Disease mostly occurs post-weaning—therefore, pigs need to be individually vaccinated.

Zinc oxide inclusion in the feed is extremely effective in preventing post-weaning enterobacterial dysbiosis and no other additive has been able to equal its effect in a commercial diet. Most countries in Europe have restricted or banned the use of zinc oxide, leading to major problems in controlling post-weaning intestinal health. A survey of south-west European producers reported a two-to-three-fold increase in mortality rates during the growing phase when compared with data prior to zinc oxide removal (SIP consultors, personal communication).

Different drivers demand that the pig industry reduces antimicrobial use, provides higher welfare, increases performance and keeps production costs low. Preventing disease and optimizing performance is paramount to achieving these objectives. Good intestinal health in the postweaning phase is the cornerstone of the prevention of the challenge of STEC and Oedema Disease. Additional measures, such as vaccination, can help those producers who cannot avoid or control it at a reasonable cost. It is worthwhile monitoring the presence of clinical and subclinical disease and quantifying its impact, as some preventive strategies may present cost-effective options, increasing performance and welfare, as well as reducing the need for antimicrobial treatments.

## Figures and Tables

**Table 2:** Different clinical presentations and commonly associated symptoms.

Clinical Presentation	Organs or Tissues Affected	Commonly Associated Symptoms and Visible Lesions *
Peracute	(General or non-specific).	Sudden death without other signs being detected and no specific lesions.
	Central nervous system.	Detection is difficult. Prostration, lack of activity, unresponsiveness.
Acute	General	Mortality up to 90%. Impaired growth.
	Central nervous system	Neurological signs: altered behaviour, reduced activity, prostration, anorexia. Impaired locomotion: incoordination, disorientation, movement in circles.
	Lungs	Breathing difficulties.
	Skin	Subcutaneous oedema, easily visible on eyelids and forehead.
	Stomach	Gastric wall oedema. Ulcers and haemorrhagic mucosa. Full stomach with dry contents.
	Intestines	Range from normal to constipation or watery diarrhoea. Traces of blood sometimes. Mesocolon oedema.
	Other	Weird squeak due to laryngeal oedema.
Subclinical	-	Impaired growth rate. Vascular necrosis and associated changes in tissues. Ear necrosis.
Chronic (lasting for several weeks).	-	Impaired growth rate for several weeks.
	Nervous and muscular.	Unilateral nervous signs; movement in circles. Tilted head. Muscular weakness. Atrophy in leg muscles.

* For practical purposes, only the most commonly associated symptoms are included, while other reported symptoms, which are less common, anecdotal or not directly associated with one or several clinical presentations in particular, have been excluded. These can be seen in Table 1. Adapted from [1,11,18,19,20,21,22,27,30,35].

**Table 3:** Reported effects of Stx2e toxoid vaccines on clinical and/or subclinical Oedema Disease in different field scenarios.

Effect	Range of Reported Improvements *	Sources (*n* = 43)
*Clinical signs of Oedema Disease*		
Reduction of mortality	Up to 100% mortality reduction on affected pigs, accounting for 1.7% to 24.23% of the pigs in the trial groups. Average = 7%.	[8,10,45,46,47,48,49,50,51,52,53,54,55,56,57,58,59,60,61,62,63,64,65,66,67]
Improvement in growth rate	Improvement in the range of 6.2 to 22.2 gr/day extra during the nursery period. Average = 17.6 gr/d. Approx. 22.68 to 93 gr/day of extra growth from wean to finish. Average = 45.7 gr/day.	[10,45,46,48,49,54,59,61,63,64,65]
Reduction of antimicrobial use	Product saving valued at 0.19 to 2.18 EUR/grower. From 2.4 fewer doses injected per pig to 3 fewer weeks on oral medication.	[47,49,53,56,57,58,61,63,64]
Clinical signs	Reports of clinical signs disappearing in most vaccinated pigs that were exposed to STEC challenge.	[10,45,51,52,59,60,65,66,67]
*Only Subclinical signs*		
Reduction in mortality	Mortality reduction ranges from 0.3 to 6%. Average = 3.1%.	[10,63,65,68,69,70,71,72,73,74,75,76,77]
Improvement in growth rate	Growth improvement of 5 to 26.5 gr/day extra during the nursery period. Average = 17.5 gr/day Improvement of 22 to 33 gr/day extra when computing the whole wean-to-finish period. Average = 26 gr/day.	[10,11,63,65,68,69,70,71,74,78,79,80,81]
Antimicrobial reduction	Reduction of 22% of the antimicrobial cost. Savings of 0.54 to 0.71 EUR/grower. Reduction of 10.3 DDD/year (Defined daily dose per year).	[63,74,75,76,77,81,82]
Better weight uniformity	3% better weight dispersion.	[78]
Better feed conversion ratio (FCR)	FCR improvement ranges from 0.9 to 0.26 in nursery and 0.12 in fattening.	[71,72,81]
Ear necrosis prevention	Ear necrosis incidence decreased from 36% to 15%.	[11]
Fewer problems with *S. suis*.	Lower clinical impact due to *S. suis.* and reduction in antimicrobial use.	[31,77,81]

(*) Outlier values caused by trial design are not considered for calculation in these figures. Values from studies combining clinical and subclinically affected pigs are not used to calculate the effect of subclinical impact.

## Data Availability

No new data were created or analyzed in this study.

## Abbreviations

The following abbreviations are used in this manuscript:

EDEC	Edema disease *Escherichia coli*
Gb3	Globotriaosyleramide
Gb4	Globotetraosylceramide
LGS	Leaky gut syndrome
MAMPs	Microbe-associated molecular patterns
SINS	Swine inflammation and necrosis syndrome
STEC	Shiga toxigenic *Escherichia coli*
Stx2e	Shiga toxin 2e
VT2e	Verotoxin 2e
VTEC	Verotoxigenic *E. coli*
Vtx2e	Verotoxin 2e
